# Will I publish this abstract? Determining the characteristics of medical education oral abstracts linked to publication

**DOI:** 10.36834/cmej.69558

**Published:** 2020-12-07

**Authors:** Jean-Michel Guay, Timothy J. Wood, Claire Touchie, Chi Anh Ta, Samantha Halman

**Affiliations:** 1 Department of Medicine, University of Ottawa, Ontario, Canada.; 2 Department of Innovation in Medical Education, University of Ottawa, Ontario, Canada.; 3 Medical Council of Canada, Ontario, Canada.

## Abstract

**Background:**

Prior studies have shown that most conference submissions fail to be published. Understanding factors that facilitate publication may be of benefit to authors. Using data from the Canadian Conference on Medical Education (CCME), our goal was to identify characteristics of conference submissions that predict the likelihood of publication with a specific focus on the utility of peer-review ratings.

**Methods:**

Study characteristics (scholarship type, methodology, population, sites, institutions) from all oral abstracts from 2011-2015 and peer-review ratings for 2014-2015 were extracted by two raters. Publication data was obtained using online database searches. The impact of variables on publication success was analyzed using logistic regressions.

**Results:**

In total, 953 oral abstracts were reviewed from 2011 to 2015. Overall, the publication rate was 30.5% (291/953). Of 531 abstracts with peer-review ratings, between 2014 and 2015, 162 (31%) were published. Of the nine analyzed variables, those associated with a greater odds of publication were: multiple vs. single institutions (odds ratio (OR) = 1.72), post-graduate research vs. others (OR=1.81) and peer-review ratings (OR=1.60). Factors with decreased odds of publication were curriculum development (OR=0.17) and innovation vs. others (OR=0.22).

**Conclusion:**

Similar to other studies, the publication rate of CCME presentations is low. However, peer ratings were predictive of publication success suggesting that ratings could be a useful form of feedback to authors.

## Abstract (fr)

**Contexte:**

Des études ont montré que la plupart des résumés soumis pour présentations orales ne sont pas ultérieurement publiés. Il pourrait être utile aux auteurs de comprendre les facteurs qui favorisent la publication. À l’aide de données provenant de la Conférence canadienne sur l’éducation médicale (CCÉM), notre objectif était d’identifier les caractéristiques des résumés permettant de prédire les chances de publication et en particulier l’utilité des cotes attribuées par les réviseurs.

**Méthodologie:**

Les caractéristiques des études (type de projet d’érudition, méthodologie, population, établissements, institutions) de tous les résumés de présentation orale soumis pour les conférences de 2011 à 2015 et les cotes attribuées par les réviseurs entre 2014 et 2015 ont été extraites par deux évaluateurs. On a obtenu des données de publication en faisant des recherches dans des bases de données en ligne. L’effet des variables sur le potentiel de publication a été examiné à l’aide de régressions logistiques.

**Résultats:**

Au total, 953 résumés ont été révisé des années 2011 à 2015. Le taux de publication était de 30.5% (291/953) en somme. Des 531 résumés ayant été évalués des pairs, entre 2014 et 2015, 162 (31 %) ont été publiés. Parmi les neuf variables analysées, celles qui ont été associées à un nombre élevé de chances de publication étaient les suivantes : projet multi-institutionnel par rapport à institution unique (risque relatif (RR) = 1,72), travaux de recherche post-graduée par rapport à d’autres types (RR = 1,81) et présence de cotes attribuées par les réviseurs (RR = 1,6). Les facteurs associés à des moindres chances de publication étaient les suivants : articles portant sur le développement de cursus (RR = 0,17) et les innovations, par rapport à d’autres (RR = 0,22).

**Conclusion:**

Comme ce fut le cas pour d’autres études, le taux de publication à la suite d’une présentation au CCME est faible. Cependant, les cotes attribuées par les réviseurs permettaient de prédire les chances de publication ce qui semble indiquer que les cotes pourraient constituer une forme de rétroaction utile aux auteurs.

## Introduction

The field of medical education research has grown exponentially since its inception in the 1950s.^1^ There has been a substantial rise in medical education journals, in the number of conferences focused on medical education, and in established programs geared specifically to this field.^2^^,^^3^ Aligning with this pattern, there has been an increased demand for formalized postgraduate qualifications in medical education with the number of offered master’s degrees in health professions education programs rising by more than 10-fold in the last 15 years.^4^^,^^5^ Clinicians with expertise in medical education research are being sought and hired for academic positions.

Productivity in academic medicine is often measured by metrics established for basic science and clinical research. Indeed, in most universities, promotion, remuneration, and tenure systems reward individuals using traditional citation-based journal rankings.^6^ This situation is not new. In a review of the literature on evaluation in higher education, Tan^7^ highlighted that research accomplishments are often based on number of publications. Today, publication in high status journals continues to be the gold standard for academic success^8^ and the mantra of ‘’publish or perish’’ remains very much alive.^9^

A common strategy used by many researchers as a step toward publication is to present their work at medical education conferences. This does not however guarantee publication, which is often referred as an indication of academic success. Despite the increasing number of attendees and presenters at such conferences, there is evidence that most submissions fail to go on to peer-reviewed publications.^10^^,^^11^ This is not unique to the field of medical education. A Cochrane review of over 29,000 abstracts presented at biomedical scientific meetings reveals a mean publication rate of 44.5%.^12^ The average rate of publication of abstracts presented at international conferences in various scientific disciplines has been found to vary widely from 8% to 81% depending on the field of research.^10^

There are potential long-term consequences to researchers not publishing. For example, publications are an important metric for academic promotion, and failure to publish completed research may lead to unnecessary duplication of work, by repeating research projects that failed to be published in the literature.^13^ Further, with growing fiscal constraints placed on faculties of medicine, some might question the benefit of attending and presenting at conferences if it is not recognized for promotion and/or does not lead to a publication.

Given the perceived benefit of presenting work at research meetings and the negative consequences of not publishing, documenting the publication rate of conference presentations is important. Past research has shown that only about 35% of presentations in the field of medical education are eventually published.^10^^,^^11^ However, this research is based on conferences that took place more than a decade ago. With the exponential growth of medical education research and the increasing attendance at research meetings, it is important to see if this pattern of publication has changed. In addition, identifying characteristics of conference presentations that lead to an increased chance of publication would also be informative.

Therefore, the goal of this study was to determine characteristics associated with publication including the predictive value of the conference abstract review process. Secondary aims included identifying the rate of peer-reviewed journal publications stemming from oral presentations of abstracts presented at a national meeting over a 5-year period, from 2011 to 2015.

## Methods

### Study design

To address our research question, we focused on the Canadian Conference on Medical Education (CCME). In Canada, CCME is the premier venue of presentation of work spanning the education continuum from undergraduate medical education to continuing professional development. Considering that Canadian medical education researchers are responsible for 37% of published research in medical education,^14^ focusing on this conference should be representative of the literature. The number of presentations each year now averages around 400 with roughly 1500 people attending. Applicants must indicate whether their abstract should be considered for an oral abstract only, poster only or could be considered for either upon submission. The number of submissions received yearly is not publicly available.

All oral abstracts from the 2011-2015 CCME conferences were considered. We chose to focus on oral abstracts for two reasons. First, prior studies have shown that oral abstracts are twice as likely to lead to publication compared to poster presentations^11^ and second, CCME does not archive abstract submissions accepted as poster presentations on a consistent basis.

To determine publication status, Medline, Embase and Google Scholar were searched using the names of the first and second authors. If the first search was negative, first and last authors were then searched, followed by key words from the title, abstract or both. In addition, conference submissions go through a rigorous peer-review process to ensure that quality work is presented at the conference and as part of this process, there are peer review ratings that can be incorporated into the study. CCME has been archiving conference abstract peer-reviewed ratings since 2014. As a subsequent analysis, the average rating assigned to each oral abstract submission from 2014 and 2015 were linked to the collected data to determine whether these ratings were predictive of publication status.

Once the data extraction was done, 20% of the extracted data was reviewed by two investigators, by doing a cross-review to ascertain standardization method of extraction between investigators. The study team felt that 20% was a reasonable portion of data to review given some evidence that omission and inaccuracy errors in systematic review data extraction ranges from 10.0 to 15.7%.^15^ Inter-rater agreement was obtained if the second reviewer was extracting the exact same data from the oral abstracts compared to the first reviewer. If inter-rater agreement was not attained, a third reviewer, with advanced knowledge and experience in research methodology, arbitrated to find a consensus.

Each oral abstract was then reviewed by one of the two investigators who extracted the following information: type of scholarship (curriculum development, innovation, program evaluation or research), research method (qualitative, quantitative, mixed methods or other), study participants (undergraduate, postgraduate, practicing physicians, international medical graduates (IMGs), interprofessional, unspecified population or other), number of study sites (single or multiple), number of institutions in the author group, main abstract theme (e.g., teaching and learning, assessment, etc.), publication status (positive or negative), publication date (if positive), journal type (medical education or other), publication type (full-length or other (which includes published abstract, perspective, opinion and commentary) and publication model (traditional or open access (defined as a free access without subscription fees for the reader or mandatory access through institutional VPN)).

The study received exemption from the Ottawa Health Science Network Research Ethics Board (OHSN-REB).

### Statistical analysis

Descriptive statistics were used to describe publication status, time to publication and publication type. We ran two multivariable logistic regression models to ascertain the effects of the various variables on publication success. The first model included all abstracts from 2011-2015 with the independent variables mentioned above. Because these were categorical variables, dummy coding was used with a total of 21 variables being entered into the model. Within each category, the variable with the highest number was used as the base reference for dummy coding. For this analysis, a backward logistic regression model was used. This type of analysis is appropriate if one is exploring data and there is no theoretical reason why one variable might be more important than another. Using data from 2014-2015, the second analysis incorporated the average peer review rating. Due to the smaller sample size and considering the redundancy in the dataset, only the significant variables identified in the previous analysis plus the CCME rating were used in this logistic regression model.

## Results

In total, 953 oral abstracts from 2011 to 2015 were reviewed, with an inter-rater agreement of 90.5% (172/190) for the 20% cross-reviewed abstracts. The overall publication rate was 30.5% (291/953). The oral abstracts presented in 2011 were published at 28.6% (32/112), 44.7% (46/103) for 2012, 24.6% (51/207) for 2013, 28.2% (74/262) for 2014 and 32.7% (88/269) for 2015. The oral abstracts’ characteristics leading to a successful publication are summarized in Table 1.

**Table 1:** Characteristics of oral abstracts

	Successful Publication	
	%	*N*
Type of Scholarship		
Research	43.8%	211/482
Program evaluation	26.1%	41/157
Curriculum development	12.7%	8/63
Innovation	12.4%	31/251
Research Methods		
Qualitative	42.9%	119/277
Quantitative	34.8%	112/322
Mixed methods	30.5%	32/105
Other methods (descriptive)	11.2%	28/249
Study participants		
Postgraduate	42.4%	53/125
Interprofessional health	39.1%	18/46
Practicing physicians	36.3%	53/146
International medical graduates	25%	5/20
Undergraduate	24.3%	63/259
Other healthcare professionals	10%	1/10
Other	32%	51/159
Unspecified/none-population based study	25%	47/188
Number of Authors		
1 author	25.4%	182/717
2 authors	44.4%	63/142
3 authors	51.8%	29/56
4 authors	45.5%	10/22
5 authors	45.5%	5/11
6 authors	50%	1/2
7 authors	33.3%	1/3
Number of Institutions on Authors list		
Multiple institutions	46.2%	109/236
Single institution	25.4%	182/717
Abstract Themes		
CanMEDS	38.7%	24/62
Assessment	37.5%	36/96
Teaching and learning	32.3%	106/328
Quality assurance	28.9%	11/38
Stage of practice	28.8%	49/170
Wellness	19.0%	8/42
Innovation	16.7%	5/30
Special populations	14.5%	11/76
Other	36.9%	41/111

The median time to publication, calculated by the conference year subtracted from the publication year, was 1 year, ranging from -1 (an oral abstract published in the year that preceded the respective CCME presentation) to 7 years. The mean time to publication and standard deviation were 1.3 and 1.32 years respectively. The characteristics of the published oral abstracts are presented in Table 2.

**Table 2:** Characteristics of published oral abstracts

	Percentage	Number of abstracts
Type of journals		
Medical education	68%	199/291
Other	32%	92/291
Length of article		
Full length	96%	278/291
Other	3.8%	11/291
Publication model		
Traditional peer-reviewed journal	75%	218/291
Open-access	25%	73/291

For the first analysis using 2011-2015 data, of the 21 predictor variables, a total of eight were statistically significant as shown in Table 3. Four variables (i.e., multiple institutions, unspecified population, postgraduate population and qualitative methods) were found to increase the odds of publication. Furthermore, four variables (i.e., curriculum development, innovation, program evaluation and other research methods) led to a decrease in the odds of publication.

**Table 3:** Odds ratio for OAs characteristics and publication status

	Odds Ratio	*P* value	95% C.I.	
			Lower	Upper
Multiple vs single institution	2.16	<0.001	1.55	3.00
Unspecified population vs all populations	1.98	0.006	1.21	3.23
Postgraduate vs all populations	1.80	0.006	1.18	2.76
Qualitative vs all other methods	1.56	0.010	1.11	2.17
Program evaluation vs all other scholarship	0.63	0.031	0.41	0.96
Other methods vs all other methods	0.43	0.009	0.23	0.81
Innovation vs all other scholarship	0.32	<0.001	0.18	0.54
Curriculum development vs all other scholarship	0.23	<0.001	0.11	0.52

For the second analysis, the eight significant variables identified above plus the 2014-2015 CCME average peer-review ratings were included. The peer-review process used by CCME involved independent review by four experts in medical education. A total of five variables were statistically significant (Table 4). Three variables, including CCME average peer-review ratings, multiple institutions, and research involving postgraduate populations were linked to successful publication odds. Two variables (i.e., innovation scholarship and curriculum development scholarship) were linked to decreased odds of publication. The remaining variables (qualitative studies, undefined population, program evaluation and other research method), extracted from the first multivariable logistic regression analysis, were not statistically significant.

**Table 4:** Adjusted odds ratio for OAs characteristics and publication status

	Odds Ratio	*P* value	95% C.I.	
			Lower	Upper
Postgraduate vs all populations	1.81	0.036	1.04	3.14
Multiple vs single institution	1.72	0.018	1.10	2.69
CCME scores	1.60	0.021	1.07	2.38
Innovation vs all other scholarship	0.22	<0.001	0.10	0.46
Curriculum development vs all other scholarship	0.17	0.001	0.06	0.48
No population vs all populations	1.79	0.107	0.88	3.65
Qualitative vs all other methods	1.34	0.208	0.85	2.11
Program evaluation vs all other scholarship	0.60	0.054	0.35	1.01
Other methods vs all other methods	0.46	0.079	0.19	1.10

## Discussion

Dissemination of scholarly work and research is important to advance a specific field and for academic promotion, but it is challenging to establish which characteristics increase the odds of eventual publication. A common strategy used by many researchers is to present their work at medical education conferences, but this may not necessary lead to publication success. In this study we considered abstracts presented at CCME to determine not only the successful publication rate, but also if we could identify any factors that would increase the likelihood of publication.

We found that, between years 2011 and 2015, 30.5% of all oral abstracts presented at CCME were published in the literature. Most studies were published within 12 months of presentation in journals dedicated to medical education. Our overall publication rate was in keeping with that reported in studies by Cheng et al. (22%) in simulation education^10^ and Walsh et al. (34.7%) in medical education.^11^ These data can be interpreted in two ways. The initial inclination may be to imply that publication rate has not increased despite the increased number of submissions, conference attendees, and medical professionals with formal degrees in medical education. However, despite the drastic increase in number of submissions to conferences, the rate of publications has remained relatively similar which means that an absolute larger number of scholarly projects have been published (from 32 published abstracts in 2001 to 88 in 2015). This could possibly be explained by the increase in journals dedicated to medical education and special education issues in other types of journals.^16^

We were able to identify conference oral abstract characteristics that led to increased odds of publication. The strongest predictor of successful publication was having multiple institutions represented in the author group. This is similar to past studies^10^ and may not be surprising given that multicenter studies have many advantages over single center studies including a potentially broader range of researcher expertise, larger and more diverse sample sizes which may make findings more generalizable and foster collaboration between sites.^17^ We also found differences in our study, compared to prior studies, which has shown that PGME population, as a research subject, was more likely than UGME to be published. Amongst research methods, qualitative research methodologies were also associated with an increased odds ratio of successful publication. There has been a call in recent years for more qualitative research in medicine in general^18^ and in medical education.^19^ The reason is that qualitative research methods can address aspects of a research question that are different than what can be found using quantitative research methods.

Oral abstracts that did not clearly specify a population and those focused on postgraduate trainees had a higher odds ratio of successful publication. Studies with ‘unspecified populations’ were typically those analyzing concepts (e.g., curricula, teaching) or utilizing data banks for research purposes. It is possible that research on data is more readily accessible than research with participants given that ethical requirements, recruitment, and availability of resources work in favor of these types of studies. It is not apparent why studies with postgraduate participants were published with a higher rate than other types of participants. There may be more journals with this target audience and there might be an interest to study this population, as post-graduate trainees are at a career turning point near their independent practice and gather assessment data from a wider variety of sources (e.g. Medical Council of Canada, Royal College of Physicians and Surgeons of Canada, Faculty of Medicine, etc.).

This study also identified characteristics that were associated with decreased odds of successful publication. Interestingly, all types of scholarship (i.e., curriculum development, innovation and program evaluation) other than what is traditionally considered education research were associated with a lower rate of publication. This may be reflective of these types of scholarship typically being more descriptive of local initiatives, which focus more on sharing experience with their peers in other institutions, as opposed to being more widely generalizable or adding to the literature. Finally, studies that did not clearly fit within traditional methodologies (i.e., descriptive study, program evaluation, innovation) were also associated with a lower rate of publication which likely reflects lack of clarity in the methodological approach used.

A subsequent analysis that incorporated these predictive variables in addition to CCME peer-review ratings found similar results, further confirming that scholarly works involving multiple institutions and focused on postgraduate education were more likely to be published. Although labor intensive, this study supports the utility of the peer-review process in identifying abstracts of ‘higher’ quality given that higher score help predict publication status.

Limitations of this study include that this review was performed on oral abstracts from a single conference rather than exploring different medical education conferences. We are however confident that CCME is representative of medical education research at large because Canadian researchers are highly published, representing 37% of the medical education literature, but publication rate may be different for other medical education conferences.^14^ We reviewed abstracts from multiple years, and by ending in 2015, we could ensure that researchers had sufficient time to publish their work. It is possible that more recently presented work may have been more successfully published but given the stability in publication rate over the last decade, this seems less likely. Although the search strategy was thorough and was done by two independent reviewers, some publications might have been missed, despite using Medline, Embase and Google Scholar. Many manuscripts had titles that differed from the original oral abstracts. Principal, second and senior authors in the author group also varied, potentially leading to not being able to capture a publication.

## Future research

Other characteristics, such as having a PhD researcher amongst the authors were not explored. Potentially PhD researchers may be more grounded in theory and could bring a perspective to a study that clinician educators may not have. PhD researchers may also be expected to publish more in the literature, in most institutions, as compared to medical students, residents or staff physicians who are usually more clinically focused. If so, collaborating with a PhD researcher should be considered for future studies as this might be another characteristic leading to publication. Unfortunately, not all oral abstracts mentioned the educational degree of their authors. Finally, while presentation at conferences may not always lead to publication, the peer-review process may still prove helpful to researchers as they may receive crucial feedback from reviewers. It would be interesting to investigate how researchers use this feedback to influence future submissions and whether it is felt to be impactful for future work. This would likely be best explored with qualitative research methodologies.

## Conclusion

In summary, while conference presentations are often considered a step towards publication, this research has demonstrated that this is not always the case with only 30.5% of CCME oral abstracts being published as full studies. We were able to identify specific characteristics that lead to increased or decreased odds of publication including the predictive value of conference peer-review scores. While presentation at conferences may not always lead to publication, the peer-review process may still prove helpful to researchers as they may receive crucial feedback from reviewers with expertise in medical education.
